# WHAT IS PAIN? A HISTORY THE PROTHERO LECTURE*

**DOI:** 10.1017/S0080440113000078

**Published:** 2013-12

**Authors:** Joanna Bourke

## Abstract

What is pain? This article argues that it is useful to think of pain as a ‘kind of event’ or a way of being-in-the-world. Pain-events are unstable; they are historically constituted and reconstituted in relation to language systems, social and environmental interactions and bodily comportment. The historical question becomes: how has pain been *done* and what ideological work do acts of being-in-pain seek to achieve? By what mechanisms do these types of events change? Who decides the content of any particular, historically specific and geographically situated ontology?

Pain does not emerge naturally from physiological processes, but in negotiation with social worlds. It is surprisingly difficult, however, to define what is meant when people use that word ‘pain’. The English noun ‘pain’ encompasses a host of phenomena that are incommensurate. ‘Pain’ is a label that adheres to scraped knees, headaches, phantom limbs and kidney stones. It is assigned to heart attacks and heartaches. The adjective ‘painful’ is so broad that it can be applied to a toothache as easily as to a boil, a burst appendix and a birth. Pain can be inflicted by knives or hula-hoops (as in the 1959 mini-epidemic of children diagnosed with ‘hula-hoop syndrome’ caused by ‘excessive hooping’).^1^

Furthermore, even a cursory examination of the historical record uncovers a headache-inducing range of theological, scientific, medical and philosophical definitions of pain. In 1882, Friedrich Nietzsche famously declared that ‘I have given a name to my pain’; it was called ‘dog’. For him, pain was ‘just as faithful, just as obtrusive and shameless, just as entertaining, just as clever as any other dog, and I can scold it and vent my bad mood on it, as others do with their dogs, servants, and wives’.^2^ It was an apt analogy, even if rather insulting to non-figurative dogs, servants and wives. However, if pain was a dog, it was a beast of gargantuan proportions. Nietzsche was adopting a functionalist definition: his pain-Dog was defined by its function in the great philosopher's life. Such ways of conceptualising pain have proliferated. For centuries, theologians have assumed that pain was a kind of chastising communiqué from a Higher Being; nineteenth-century evolutionists contended that it was a mechanism to protect the organism from harm; and many clinicians from the late nineteenth century onwards have drained pain of any intrinsic meaning altogether, making it little more than a sign or symptom of something else (a dis-ease). With brain imaging technologies from the late twentieth century, the subjective person-in-pain could be eradicated altogether, with pain morphing into little more than ‘an altered brain state in which functional connections are modified, with components of degenerative aspects’.^3^

Still others have diced pain using different scalpels. In innumerable ways, philosophers and scientists have sought to pare pain back to its bare skin and bones. Was pain the reaction of filaments and animal spirits to noxious stimuli, as René Descartes and his disciples believed in the seventeenth century and beyond?^4^ Was it caused by ‘too great irritability’ or ‘a want of sufficient irritability’, as the author of *Asthenology* (1801) claimed?^5^ Or was it more correct to say that pain was a sensation in the sense that it ‘has a threshold, is localised and referred to a stimulus’?^6^ In the 1830s, Sir Charles Bell in England and Francois Magendie in France focused on the biological nature of pain in the context of the motor and sensory functions of the dorsal (Bell) and ventral (Magendie) roots of the spinal cord. Johannes Müller, Richard Bright, Maximiliary Von Frey and A. Goldschneider fixed attention on the nerves, disagreeing fiercely about whether specificity theory (the body has a separate sensory system for perceiving pain) or pattern theory (the receptors for pain are shared with other senses such as touch) best described the physiology of pain.^7^ In 1976–7, the International Association for the Study of Pain (IASP) attempted to tame Nietzsche's beast by calling together a diverse group of pain specialists (including experts in neurology, neurosurgery, psychiatry, psychology, neurophysiology, dentistry and anaesthesia) to adjudicate definitively on the question ‘what is pain?’ Their definition is now the most cited one in the field of pain studies. The IASP concluded that pain is ‘an unpleasant sensory and emotional experience associated with actual or potential tissue damage, or described in terms of such damage’. This definition emerged directly from the invention in 1965 of the Gate Control Theory of Pain, which introduced the idea of a ‘gating mechanism’ in the dorsal horns of the spinal cord that allowed the perception of pain to be modified. Crucially, the Gate Control Theory and, consequently, the IASP's definition insist that sensory, cognitive and affective processes influence people's experience of pain. As such, the definition is remarkably flexible and it opens the door to social, psychological and physiological explorations. This does make it very useful indeed to the historian.

However, as historians have consistently argued, it is extremely problematic to overlay a late twentieth- and early twenty-first-century understanding of pain on to earlier periods. Equally troubling, adopting the IASP's definition would have meant taking a particular position on that long-standing, thorny debate about what some have dubbed the ‘myth of two pains’,^8^ that is, emotional versus bodily pain. Although the IASP's definition may seem to side with those who seek to undermine the distinction between the emotional and the physiological, in fact it does nothing of the kind. It simply states that both are valid ‘pains’ (if a person described her emotional pain in terms of tissue damage, it is allowed to be called ‘painful’). The Cartesian distinction between mind and body (and it may be noted that Descartes himself was not a fully signed-up Cartesian)^9^ is alive-and-well and does a vast amount of ideological work for physicians, psychiatrists, psychologists, the pharmaceutical industry and chronic pain patients today.

One useful starting point in beginning to define what is meant by the word ‘pain’ can be found in the musings of a prominent Victorian physician, Dr Peter Mere Latham. Latham had been born in London in the year of the French Revolution and died eighty-six years later. He was one of the most renowned physicians in London, working at the Middlesex Hospital and then St Batholomew's, and (like his father) was appointed Physician Extraordinary to the Sovereign. Portraits show him bedecked in robes, with a magisterial forehead, penetrating gaze and self-assured smile. Given that his everyday routines were frequently shattered by attacks of asthma, he clearly had thought a great deal about suffering.

What is most striking about Latham, however, are his thoughts on bodily pain, published between the 1830s and the early 1860s. He also wanted to know the answer to that simple question: what is pain? Latham recognised that pain assumed many guises. ‘There is a Pain which barely disturbs the complacency of a child’, he noted, and ‘a pain which is too much for the strength of a giant’. Are these two kinds of pain actually the same, differing only ‘in degree’? Could it really be the case that ‘the smallest Pain contain[s] all that essentially belongs to the greatest, as the minutest atoms of matter have separately the same properties of their largest aggregates’, he asked? In everyday language, dramatically different experiences of pain are spoken of using one word – ‘pain’. But if we ‘suppose ourselves at the bed-side and within hearing, when Pain raises its cry of importunate reality’, the likenesses of painful experiences are exposed as nothing more than a linguistic deceit. The ‘things of life and feeling’ – that is, each person's unique encounter with suffering – are ‘different from all things in the world besides’.^10^

So, how did Latham seek to define pain? The correct response to anyone who asks ‘what is Pain?’, he rather grumpily contended, was simply to state that ‘he knew himself perfectly well what it was’ and he ‘could not know it the better for any words in which it would be defined’. Hammering home the point, Latham insisted that ‘Things which all men know infallibly by their own perceptive experience, cannot be made plainer by words. Therefore, let Pain be spoken of simply as Pain.’^11^

Latham's definition of pain – pain is what is spoken about as ‘Pain’ – is one that many historians, anthropologists, sociologists and even clinicians espouse. Anyone claiming to be ‘in pain’, *is* in pain; if a person describes her experiences as ‘painful’, they are. For the purposes of historical analysis, so long as someone says that they are suffering, that claim is accepted. In Latham's words, ‘The fact of pain being suffered at all must always be taken on the patient's own shewing [*sic*].’ Of course, like Latham, we might admit that ‘there is such a thing as shamming Pain’,^12^ but that does not alter our primary definition.

This approach to pain has been highly productive. It is well suited to the way many historians conduct their research. It is profoundly respectful towards the ways peoples in the past have created and recreated their lives, and it remains courteously neutral about the veracity of any specific claim. It allows for multitude, even conflicting, characterisations of suffering. Crucially, the definition enables historians to problematise and historicise every component of pain-talk. It insists that ‘pain’ is constructed by a host of discourses, including theological, clinical and psychological ones. Done badly, it can lead to literary practices that assume that ‘pain’ can be ‘read’ transparently from various texts; done well, however, this approach to pain encourages subtle, deconstructive analyses of past experiences and behaviours.

However, the definition comes up against a major limitation. The clue to the problem lies in the fact that whenever Latham wrote about ‘pain’, he capitalised it: for Latham, pain was Pain. In other words, there is an assumption that pain is an ‘it’, an identifiable thing or concept. To be fair, Latham recognised this problem. He was not convinced that ‘pain’ was an ‘it’, excusing himself on the grounds that his reifying (although he would not have used that word) of ‘Pain’ was driven by pragmatic observations. As he observed, ‘No man, wise or foolish, ever suffered Pain, who did not invest it with a *quasi* materialism.’ In the throes of physical anguish, even the most rational philosopher finds himself ‘outreasoned by his feelings’. ‘I have known many a philosopher’, Latham continued, ‘take to rating and chiding *his Pain*, as if it were an entity or quiddity of itself.’ Therefore, ‘for practical purposes, we must often let people think and speak of things as they seem to be, and not as they are, making a compromise between philosophy and common sense. We must let them speak so of Pain. There is no help for it.’

Ignoring Latham's condescending tone, his basic point is a legitimate one. Sufferers of pain are entitled to say ‘I don't know what *you* mean by pain, but *I* know “it” when I feel “it”’, and then go on to describe their pain as though it were an independent entity within their body (‘I have a pain in my tooth’) or an entity that attacks from the outside (as in: pain is a weapon that stabs, a fire that burns, an animal that bites). But, for the historian, there are many dangers involved in referring to pain as though it were an entity. The chief one is that it risks giving ‘pain’ an independent life. The ease with which we can slip into making this error can be illustrated by turning to Elaine Scarry's influential book *The Body in Pain* (1985). Scarry argues that pain is outside of language, absolutely private and untransmittable. Indeed, in her most often-quoted statement, Scarry goes even further, insisting that ‘Physical pain does not simply resist language but actively destroys it, bringing about an immediate reversion to a state anterior to language, to the sounds and cries a human being makes before language is learned.’^13^ This is an extreme version of reification. As literary scholar Geoffrey Galt Harpham rightly observes, such an argument

treats as an immediate and monochrome physical experience, a baseline of reality, what is in fact a combination of sensations, dispositions, cultural circumstances, and explanations, a phenomenon involving body, mind, and culture. She has, in other words, misconceived the character of pain precisely by giving it a character, by treating it as a fact – a brute fact, the first and final fact – rather than as an interpretation.^14^

In other words, Scarry has fallen into the trap of treating metaphoric ways of conceiving of suffering (pain bites and stabs; it dominates and subdues; it is monstrous) as descriptions of an actual entity. Of course, metaphorically, pain is routinely treated as an independent entity within a person, as in statements such as ‘he has a pain *in* his shoulder’, ‘the pain *went away*, and ‘bleeding will *get rid of* the pain’ but, for Scarry, these metaphors are literalised.^15^ ‘Pain’, rather than a person-in-pain, is given agency. This is an ontological fallacy.

How can pain be conceived of non-ontologically? It is helpful to begin from the premise that pain is not an object; it is a *type of event*. Pain is a *way* of being-in-the-world. Pain-events are unstable; they are historically constituted and reconstituted in relation to language systems, social and environmental interactions and bodily comportment.

What is meant by saying that pain is an event? By designating pain as a ‘type of event’ (I will discuss what I mean by ‘*type of* event’ shortly), I mean that it is one of those recurring activities that we regularly experience and witness that participates in the constitution of our sense of self and other. Events are not entities or mental objects; they are ways of being-in-the-world. An event is designated ‘pain’ if it is identified as such by the person claiming that kind of consciousness. Being-in-pain requires an individual to give significance to this particular ‘type of’ being. This word ‘significance’ is not being used in the sense of ‘importance’ (a pain can be a momentary pin-prick) but in the sense of ‘recognised’ (a stomach*ache* rather than the gurgling of the stomach before lunch). It is never neutral or impersonal. In other words, a pain-event possesses what philosopher Paul Ricoeur called a ‘mineness’.^16^ A pain-event always belongs to the individual's life; it is a part of her life story. In this way, the person becomes or makes herself into a person-in-pain through the process of naming.

An individual has to name pain – she has to identify it as a distinctive action – for it to be labelled a pain-event. But how do people know what to name as pain? If the words we use for sensations are private or subjective, then how do we know how to identify them? How do people give the label ‘pain’ to one subjective sensation and not another?

In recent years, scholars exploring the senses have turned to the ideas of the philosopher Ludwig Wittgenstein for stimulation. In *Philosophical Investigations*, Wittgenstein turned his mind to the question of whether there can be such a thing as a private language. How do ‘words *refer* to sensations’, he asked? Like Latham, he acknowledged that people routinely talk about their sensations. As Wittgenstein put it ‘don't we talk about sensations every day, and give them names’, so why the fuss? Simply put, he continued, the problem is ‘how is the connection between the name and the thing set up? This question is the same as: how does a human being learn the meaning of the names of sensations? – of the word “pain” for example.’ Wittgenstein modestly suggested ‘one possibility’, that is, ‘words are connected with the primitive, the natural expressions of the sensations and are used in their place. A child has hurt himself and he cries; and then adults talk to him and teach him exclamations and, later, sentences. They teach the child new pain-behaviour.’ He imagined an interlocutor interrupting him with the question, ‘So you are saying that the word “pain” really means crying?’ ‘On the contrary’, Wittgenstein continued, ‘the verbal expression of pain replaces crying and does not describe it.’^17^

Imagine, he mused, a world in which there were no outward expressions of sensations – where, for instance, nobody cried or grimaced. In such a world, how could a person know he was in pain? This man could scrawl an ‘S’ in his diary each time he experienced a particular sensation. But how would he know that it was the same sensation he was experiencing each time? And how would other people know what ‘S’ stood for? This diarist would have no criterion for knowing when he was experiencing ‘S’ and when ‘T’. To have any meaning, Wittgenstein concluded, words for feeling-states like pain must be inter-subjective and able, therefore, to be learned. In other words, the naming of a ‘pain-event’ can never be wholly private. Although pain is generally regarded as a subjective phenomenon – it possesses a ‘mine-ness’, to use Ricoeur's term – ‘naming’ occurs in public realms.

Wittgenstein clearly enjoyed imagining other worlds. On another occasion, he invented a world in which everyone possessed a box, which contained a beetle. No one was able to peer into anyone else's box, however. Because people only knew what the beetle was by looking into their private box, it was entirely plausible that each person believed that ‘beetle’ referred to a complete different entity. Indeed, the ‘beetle in the box’ might change regularly. The box might even be empty. But if everyone believed that they possessed a ‘beetle in a box’, then the word ‘beetle’ was useful in communication. In terms of language, in other words, the ‘actual content’ of the box does not matter. What is important is the role of the ‘beetle in the box’ in terms of public experiences.

Now substitute the word ‘pain’ for ‘beetle’: it does not matter that I have no direct access to your subjective consciousness, so long as we have a shared language to discuss our various ‘pains’. For my purposes, this ‘beetle in the box’ analogy is not perfect – after all, my pain is not ‘in’ my body (although we often speak as though it is) in the same way that a beetle is ‘in’ a box – but Wittgenstein's language-game draws attention to an approach to pain that can be very productive for historians. As Wittgenstein succinctly put it, ‘mental language is rendered significant not by virtue of its capacity to reveal, mark, or describe mental states, but by its function in social interaction’.^18^ For historians, it is important to interrogate the *different* language games that people residing in the foreign kingdom of the past have played, in order to enable us to make educated guesses about the diverse and distinctive ways people have packaged their ‘beetle in the box’ or pain in their lived-bodies.

My approach to pain also states that pain is a ‘*type of* event’. In other words, it is useful to think of pain-events in adverbial terms. Philosopher Guy Douglas has an interesting way of explaining this point. He points out that there is a difference, for example, in saying ‘I feel a sharp knife’ and ‘I feel a sharp pain.’ In the first instance, the knife is what linguists call an ‘alien accusative’ (that is, the knife refers to the object of the sentence) while, in the second instance, pain is a ‘connate accusative’ (it qualifies the verb ‘to feel’ rather than being a sensory object in itself). In the first sentence, we are ‘describing a knife *apart from* the way it feels, while in saying that *pain is sharp* we are describing the feeling’, that is, a sensation similar to being injured by a sharp object. In other words, in saying ‘I feel a sharp pain’, we are qualifying a verb rather than a noun.^19^

The other way of expressing this is by saying that pain describes the *way* we experience something, not *what* is experienced. For example, we say that a tooth is aching, but the ache is not actually the property of the tooth but is our way of experiencing or perceiving the tooth. As Douglas put it, ‘sensory qualities are a property of the way we perceive the object rather than the object itself’. Pain is ‘not the thing or object that one is feeling, it is what it is like to feel the thing or object’. Crucially, pain is not an intrinsic quality of raw sensation; it is a way of perceiving an experience.^20^ Pains are modes of perceptions: pains are not the injury or noxious stimuli itself but the way we evaluate the injury or stimuli. Pain is a way-of-being in the world or a way of naming an event.

The historical question, then, becomes: how has pain been *done* and what ideological work do acts of being-in-pain seek to achieve? By what mechanisms do these types of events change? As a type of event, pain is an activity. People do pain in different ways. There is no decontextual pain-event: so-called ‘noxious stimuli’ may excite a shriek of distress (corporal punishment) or squeal of delight (masochism); there is no necessary and proportionate connection between the intensity of tissue damage and the amount of suffering experienced since phenomena as different as battle enthusiasm, work satisfaction, spousal relationships and the colour of the analgesic-pill can determine the degree of pain felt; and people have no difficulty using the same word ‘pain’ to refer to a flu injection and an ocular migraine.

Although each individual is initiated into cultures of pain from birth, being-in-pain is far from static or monochrome, which is why it requires a history. People can – and regularly do – challenge dominant happenings of pain. Indeed, the creative originality with which some people-in-pain draw on and transform language games, environmental exchanges and bodily performances (or gestures) of suffering is striking. Of course, the most dominant ‘doing’ of pain is to objectify it as an entity – giving it independence outside the person doing the pain. It becomes important to ask, therefore, who decides the *content* of any particular, historically specific and geographically situated ontology? What is excluded in these power-acts?

As historians, it is therefore possible both to let ‘people think and speak of things as they seem to be’, as Latham expressed it (that is, conceiving of pain as an ‘it’ or an entity to be listened to, obeyed or fought) and to acknowledge that ways of being-in-pain involve a series of agents, all of whom are immersed in complex relationships with other bodies, environments and linguistic processes. It would be disingenuous to suggest that Latham would wholly agree with this interpretation, but he seemed to be gesturing towards such a position when he shrewdly remarked that

Pain, itself a thing of life, can only be tested by its effects upon life, and the function of life. And whether it be small or great (so to speak), or of whatever degree, it is to its affect upon life and the functions of life that we must look.^21^

In other words, pain is always a ‘being-in-pain’, and can only be understood in relation to the way it disrupts and alarms, authenticates and cultivates, the ‘states of being’ of real people in the world.

There are a number of advantages to adopting an events-based approach to pain in history. The first one is that we do not have to jettison Latham's main insight that ‘Things which all men know infallibly by their own perceptive experience, cannot be made plainer by words. Therefore, let Pain be spoken of simply as Pain.’ In other words, pain is what people in the past said was painful. We are not required to privilege one, historically specific meaning of pain over any other. Defining pain as a ‘type of event’ remains neutral about the ‘truth value’ of any philosophical or scientific definition. Instead, it asks: what does the scientific *content* of any particular, historically specific and geographically situated scientific ontology tell us about the way philosophers, scientists and physicians have sought to classify pain-events. As historians of science have reiterated time and again, scientific practice itself is social action. We invent, rather than discover, pain. Pain-as-event enables us to avoid reifying pain in terms of a single incarnation.

If the first advantage in thinking about pain as a ‘type of event’ is that it is historically flexible, the second advantage is that it is historically complex. People perceive pain through the prism of the entirety of their lived experiences, including their sensual physiologies, emotional states, cognitive beliefs and relational standing in various communities. As a consequence, the definition is sceptical of any pain-account that claims that pain is simply a sensual response to noxious stimuli or – to put it in the language used earlier – that Nietzsche's pain-Dog only *reacts* to the world rather than *responds* to it (which many philosophers believed was what distinguished animals from humans).^22^ As mentioned earlier, the most famous conceptualisation of ‘pain-as-ensation’ was that of Descartes. According to him, pain occurred when fast-moving particles of fire rushed up a nerve fibre in the foot towards the brain, activating animal spirits which then travelled back down the nerves, causing the foot to move away from the flame. In this model, the body was a mechanism that worked ‘just as, pulling on one end of a cord, one simultaneously rings a bell which hangs at the opposite end’.^23^ Although nociceptive impulses and endorphins were subsequently substituted for filaments and animal spirit, Descartes's basic, mechanistic model of pain-as-sensation has dominated both scientific and ‘folk’ beliefs about pain into the mid-twentieth century.

Claims that pain is simply a sensual or physiological response to noxious stimuli cannot account for the way that people in the world experience what they call pain. The model of pain-as-sensation implies that a person ‘feels’ a noxious stimuli, *after which* affective, cognitive and motivational processes ‘kick in’ – responding and interpreting the happening. However, these things occur in dynamic interaction.

Conceiving of pain as a ‘type of event’ allows us to disentangle pain-situations from pain-experiences. This is not to deny that a person dying of the plague is likely to have an excruciating headache. But many aches and pains are not caused by bodily damage. People can suffer, yet be lesion-free, as in many chronic pain states. They can be in pain, yet not possess the limb that is ‘feeling pained’, as in phantom limb sensations. They can be in situations that self-evidentially warn of agony, yet be calm. As Edmund Burke noted, Italian theological and astrologer Tommaso Campanella, who was tortured on the rack, ‘could so abstract his attention from any suffering of his body, that he was able to endure the rack itself without much pain’. As Burke correctly concluded, ‘our minds and bodies are so closely and intimately connected, that one is incapable of pain or pleasure without the other’.^24^ Being-in-pain is multifaceted: attitudes, motivations, belief systems and cognition all contribute to making or signifying the event.

Another way of making this point is to argue that pain only exists in the act of evaluating it. Not all ‘acts’ are ‘events’. Indeed, some acts (having a limb blown off in combat, to take one example) may not be designated pain-events by the person affected. A particularly stark example of this can be seen in the context of the Second World War. Lieutenant Colonel Henry K. Beecher, who served in combat zones on the Venafro and Cassino fronts, was struck by the fact that many severely wounded men did not complain of pain. Medical officers found that there was no necessary correlation between the size and depth of any specific wound and men's expressions of suffering. Beecher decided to explore this paradox systematically, questioning 215 seriously wounded men. To his surprise, three-quarters did not report experiencing significant pain. One third claimed to be feeling no pain at all, while another quarter said they were experiencing only slight pain. Remarkably, three-quarters of all seriously wounded men did not even ask for pain relief, despite the fact that being asked the question would have served as a reminder that relief was available. What was happening? Perhaps, Beecher speculated, men who had been wounded were simply less sensitive generally. But this explanation failed to account for the fact that ‘a badly wounded patient who says he is having no wound pain will protest as vigorously as a normal individual at an inept venipuncture’. Instead, Beecher found, there must be a difference between wounds caused in civilian contexts (a car accident, for example) and those caused during combat. Perhaps the strong emotions aroused in combat were responsible for the absence of acute pain. Pain might also be alleviated by the fact that wartime wounding would release a soldier ‘from an exceedingly dangerous environment, one filled with fatigue, discomfort, anxiety, fear and real danger of death, and gives him a ticket to the safely of the hospital. His troubles are about over, or he thinks they are.’ This was in contrast to civilian accidents, which only heralded in ‘the beginning of disaster’.^25^

This is not to deny the importance of the sensory nature of pain – pain is ‘what hurts’. However, it is to insist that, by itself, the sensation approach is much too narrow. The ‘sensations’ definition simply does not help explain the vast number of different sensations that we place under that single label ‘pain’ (a headache and a heartache). It also cannot help interpret the lives of people who have been lobotomised – an operation performed on people with intractable pain from 1943. These patients could still claim to be in pain (and could discriminate between different degrees of noxiousness) yet were completely uninterested and unconcerned about the sensation.^26^ The event of being-in-pain is evaluative. It stands in relation to the individual in an adverbial sense: pain is ‘not the thing or object that one is feeling, it is what it is like to feel the thing or object’.^27^ Pain may be rendered significant because it is unpleasant but there is no phenomenological state that is in and of itself ‘painful’, as any zealous saint or keen sadomasochistic practitioner can attest.

The event-ness of pain also draws attention to the fact that different emotional reactions adhere to pain-events. Depending on the presence of other objects and people, pain-events can elicit distress (face-to-face with a torturer), fear or panic (crashing through the car window), anticipation or surprise (the moments after a heart attack). They can also elicit pride (gout in the eighteenth century), relief (self-cutters) or joy (childbirth).

This can be illustrated by looking at the way Joseph Townend wrote about pain in the middle of the nineteenth century. For this impoverished manual labourer, pain was an event that adhered to some acts and not others. It was not mentioned, for example, when he wrote about the lingering deaths of four of his siblings (his father simply exclaimed, ‘Bless the Lord, there's *another safe* landed!’). Nor did he evoke pain when he described extreme hunger or working (from the age of seven) seventeen-hour shifts in the carding room of a Lancashire cotton mill. Rather, Townend only summon the spectre of pain in the context of a severe burn he suffered as a child, which resulted in his entire right arm being fused to his side. Even in this context, the process of hurting was a positive one: as he declared, ‘heaven must recompense our pains’.

For Townend, painful happenings constituted his place both in *this* world and the next. In his narration, the most important event in his entire life occurred when he trudged all the way to the Manchester Infirmary to have his arm cut free. The surgeon gruffly warned: ‘Now, young man, I tell you, if when you feel the knife, you should jerk, or even stir – you will do it at the hazard of your life.’ Anaesthetics like chloroform would not be invented for another twenty-three years and no analgesic (like whiskey or laudanum) was offered. ‘All was still’, Townend recalled, when ‘with a forcible thrust, through went the knife, as near the pit of the arm as possible, and close to my side . . . the progress of the instrument I distinctly heard’. The pain was ‘most exquisite’. As the ‘smoking wound’ was being dressed and bound, Townend was left to reflect on ‘my past neglect and wickedness in resisting the Holy Spirit’. He thought of the Methodist chapel, attendance at which he had neglected. He ‘wept bitterly’: saying his ‘mind [was] fully made up to be entirely the Lord's when I should return home’.

For Townend, bodily agony was a gift inflicted by a loving, heavenly Father. But he was equally clear that the function of pain was to teach him submission not only to hierarchies of power in the next life, but also in *this* one. Townend had only praise for his surgeon but when he attempted to thank him by shaking his hand – obviously, using his left hand – the surgeon shouted at him: ‘Do you offer a gentleman your left hand?’, then he grasped Townend's right arm and dragged him off the bed: ‘Immediately my leg and foot were covered with blood; and on the web [of skin] being loosed, I saw that it was turned black: and my poor side was drenched in blood, and smoked almost like a kiln.’ Another doctor observed that his wound was inflamed, forcing Townend to admit that he had ‘partaken rather freely of port wine’. The doctor was ‘very much grieved; and [so] he suddenly jerked up my shoulder, which made me sweat with pain, and it cracked like the firing of a pistol’. Townend's only comment on this act of the doctor whom he called ‘easy, kind, careful and communicative’ was ‘So much for wine.’ Pain was a legitimate punishment for socially insulting a ‘gentleman’ and partaking in alcoholic beverages.^28^

Pain performed two acts for Townend: it drew him into the exulted embrace of God's family while reminding him of his lowly status in the family of Man. His being-in-pain was a world away from later, secular beings, in which pain was no longer conceived of as an entity that had to be passively endured or embraced. Rather, it was an ‘enemy’ to be fought and ultimately defeated. In the words of the anonymous author of ‘The Function of Physical Pain’ (1871), now that pain had been ‘made optional’ by anaesthetics, it ‘necessitates a complete revisal [*sic*] of the theories of the purposes of bodily pain hitherto held by moralists’.^29^ When the pharmaceutical possibility of eradicating acute pain was limited, endurance could be valorised as a virtue: the introduction of effective relief (at least for the kinds of pain that plagued the young Townend's life) made passive endurance perverse rather than praiseworthy. Stripped of its mysticism and its history in foundational theological texts, pain became an evil in itself, unequally distributed (afflicting the saintly as carelessly as the sinner) and serving, at most, a rather limited diagnostic function.

In other words, people in the past interpreted their pains not as contained (in the Wittgensteinian sense of ‘the beetle in the box’), isolated, individual bodies, but in interaction with other bodies and social environments. Cognition also mattered. It made a difference whether the person-in-pain conceived of the event as being inflicted by an infuriated deity, due to imbalance in the ebb and flow of humours, an inevitable punishment for a lifetime of ‘bad habits’, or the result of an invasion by a germ.^30^ The body is never pure soma: it is configured in social, cognitive and metaphorical worlds.

This discussion anticipates the third advantage to conceptualising pain as a ‘type of event’: there is no such thing as a private pain-event. I have already discussed this aspect from a Wittgensteinian point of view, but here I mean something less philosophical and more historical. From the moment of birth, infants were initiated into cultures of pain. What these infants in the 1760s learned about the cognitive, affective and sensory meanings arising from the interface between their interior bodies and the external world was very different to what their counterparts in the 1960s learned. In the humoral physiology of the eighteenth century, for example, bodies consisted of four fluids – phlegm, black bile, yellow bile and blood. Linked to these humours were personality types (sanguine and melancholic). There were also three kinds of spirits, which acted on the humours: the natural, the vital and the animal. In this model – unlike the biomedical one that was dominant until the 1960s – distinctions between bodies, minds and souls were not clear-cut. Pain was the result of disequilibrium or imbalance. Illness was the result of disrupted relationships as much as disrupted physiologies. As a result, humoural theory provided rich figurative languages of ebbs and flows. For example, take John Hervey's 1731 description of his sister's suffering. She was

choked with phlegm, tormented with a constant cough, perpetual sickness at her stomach, most acute pains in her limbs, hysterical fits, knotted swellings about her neck and in her joints, and all sorts of disorders, consequent to a vitiated viscid [*sic*] blood, which, too glutinous and weak to perform its proper circulation, stops at every narrow passage in its progress, causes exquisite pains in all the little, irritated, distended vessels of the body, produces tumours in those that stretch most easily, and keeps the stomach and bowels constantly clogged, griped, and labouring, by the perspirable matter reverting there for want of force to make its due secretions and evacuate itself through its natural channels in the habit and the pores of the skin.^31^

Pain in this account was a blockage of natural flows. It pervaded all parts of the body, and not just particular organs. This was a world away from the body-in-pain of nineteenth-century biomedicine or twenty-first-century neurology. Physiological models of the body draw attention to certain things and not others, fundamentally affecting what is *noticed* – and given meaning – and what is regarded as incidental. The physiological body is constituted by the figurative languages that bring the body into the world. Figurative languages ‘disclose’ our being-in-the-world.^32^

Once taught what constituted a pain-event, messages communicated though language, facial expressions and gestures helped inform people-in-pain how they ought to respond. There was a rich corpus of texts explicitly instructing people how they *ought to* behave when in pain. These ‘comportment manuals’ or prescriptive pain-texts changed from the explicitly hagiographical ones of the eighteenth and nineteenth centuries to the more psychologically infused texts of more recent decades. More subtle instructions were given through gesture. There is a vast literature documenting the different ‘gestural styles’ in pain-instructions.^33^

These communicative acts were normative. They did not simply document the various ways people-in-pain responded to their affliction: they contained veiled instructions on how people *should* act. People-in-pain sought to conform to these instructions for numerous reasons. Correctly adhering to highly esteemed scripts might increase a person's confidence in an affirmative posthumous existence. Pain-performances might be important in order to protect the witnesses, rather than the sufferer herself. This was what was being conveyed in Rachel Betts's memoir of 1834. Betts was described as suffering ‘excruciating pain’, after which she observed her sister weeping. Betts was mortified, admitting that ‘I cannot help expressing how great my pain is’ since ‘it seems a relief’ to vent it. However, she added, ‘I do not wish to distress you.’ A short time later, when her mother asked her if she ‘continued easier’, Betts simply replied, ‘Quite easy.’^34^ Sufferers might also seek to conform for non-reflexive reasons (this might be especially true of those figurative ways of speaking about pain that were internalised from infancy or were deeply embedded in language).

In this way, pain can be seen as a learned exegesis. As influential pain-psychologist Ronald Melzack discovered in the 1970s, Scottish terriers who had been raised in isolation from birth and protected from all normal environmental stimuli, including painful ones, proved unable of identifying and responding ‘normally’ to a flame or pinpricks when exposed to it in maturity.^35^ They simply hadn't ‘learned’ what it meant to be-in-pain.

Of course, we do not need dogs (whether they belong to the scientist Melzack or the philosopher Nietzsche) to show us that pain is social action. Human bodies in pain are profoundly connected and communicative. Not surprisingly, the social norms expected in the expression of pain differed according to the gender, class, occupation and age of the person-in-pain. They have changed dramatically over time as people-in-pain creatively perform pain.

As a public ‘type of event’, being-in-pain was always political. Both chronic and acute beings-in-pain could be the *result of* economic deprivation (hazardous working conditions, lack of medical insurance, the failure of physicians and pharmacists in poor areas to stock the most effective analgesics) as well as the *cause of* destitution. The politics of gender also adhered to pain-events: the exquisite sensitivity required of upper-class men in the salons of Edinburgh in the late eighteenth century can be contrasted to the burly hardiness of American frontiersmen. In Martin Pernick's insightful book *A Calculus of Suffering. Pain, Professionalism, and Anesthesia in Nineteenth Century America* (1985), he correctly maps out the way that American physicians ranked different people's sensitivity to painful stimuli: in that great Chain of Feeling, certain people (distinguished by class, gender, ‘race’, religion, occupation and so on) were relatively insensitive.^36^ What he does not explore is the contradictory assumptions behind the Chain of Feeling. For instance, in nineteenth- and much of the twentieth-century discourse, non-European peoples and workers could be denigrated as possessing lesser bodies: their position at the lower echelons of the great Chain of Feeling was due to their physiological *insensibility*. However, often in the same text, they could also be designated as inferior on precisely the opposite grounds: *excessive* or ‘exaggerated’ sensitivity. The alleged insensitivity of non-Europeans, immigrants and workers was proof of their rudimentary nervous systems and thus humble status, yet the profound sensitivity of these same people was also proffered as evidence of their inferiority (they lacked strength of will).^37^ The fact that *both* beliefs could be held simultaneously was responsible for the appalling high levels of underestimation of the bodily pains of certain groups of patients. Indeed, it took until the late twentieth century for the routine underestimation of the pain of African-Americans, immigrants, women and the poor to be deemed scandalous in the medical literature.^38^ Interestingly, this vast debate was conducted almost solely in terms of the under-*medicalisation* of pain – a significant indicator about the ideological labour being performed in clinical pain-narratives.

The instability of pain-events as refracted through social interactions can also be illustrated by exploring the dramatic *shifts* in the Chain on Feeling. It was not only a contradictory hierarchy; it was also volatile over time. One illustration of this can be seen in medical discussions about the sentience of infants, which shifted from an emphasis on the exquisite sensitivity of infants in the eighteenth century to almost total insensibility to pain from the 1870s and then back again to acute sensitivity from the 1980s. The exquisite sensitivity of infants to painful stimuli was at the heart of eighteenth-century debates within the professionalisation of pediatrics – indeed, it helped perform the work of professionalisation. In the 1780s, Michael Underwood (the first obstetrician to be appointed to the Royal College of Physicians in London and the physician most responsible for establishing paediatrics as a discipline in its own right) argued that the chief reason that very young children had been neglected by the medical profession was because they lacked the capacity ‘to give account’ of their pain. As a result, their care had been entrusted to ‘old women and nurses’. It was time that this changed, he insisted. After all, infants displayed their aches and pains ‘plainly and sufficiently’ on their faces. ‘Every distempter’, he continued, had ‘a language of its own’ and it was ‘the business of a physician to be acquainted with it’.^39^ In Pye Henry Chavasse's textbook *Advice to a Mother on the Management of her Children, and on the Treatment on [sic] the Moment of Some of their More Pressing Illnesses and Accidents*, he further reflected on the ‘sympathy . . . in the nervous system’ between different parts of the infant's body. When we consider ‘how susceptible the young are to pain’, he observed, ‘no surprise can be felt at the immense disturbance and the consequent suffering and danger frequently experienced by children while cutting their *first* set of teeth’.^40^

The exquisite sensitivity of infants to painful stimuli was disrupted from the 1870s in particular. Experimental embryology, in particular, was drawing conclusions about the biology of sentience. The work of Paul Emil Flechsig was especially important. Flechsig had systematically examined sections of the brain of foetuses, newborn infants and older infants, showing that nerve fibres developed at different rates. At Flechsig's 1894 address at the University of Leipzig (published the following year as *Gehirn und Seele*), he argued that

The structures at the base of the brain, for the most part, and the cerebellum were found to be myelinated before birth; whereas in the newborn infant the cerebrum only exhibited isolated regions of myelination around the primary fissures – namely, the central, calcarine, and the Sylvian; these are the regions of the primary projection centres of movement and of the special senses. The remainder of the cortex is not myelinated, and constitutes the association centres as yet unprepared for function. Upon anatomical grounds, therefore, it may be postulated that a child at birth may have a simple sensation.^41^

In other words, at birth, infants were not fully ‘wired’.

This pain-work was much more than a rhetorical or scientific exercise: it justified giving children as old as ten inadequate pain relief – or withholding it altogether. As the author of *Modern Surgical Technique* (1938) claimed, ‘no anesthetic is required’ when carrying out even major operations (such as amputations and heart operations) on young infants. Indeed, ‘a sucker consisting of a sponge dipped in some sugar water will often suffice to calm the baby’.^42^ As late as the 1970s, over half of children aged between four and eight years who had undergone major surgery – including amputations – in American hospitals received *no* medication for pain.^43^ Dismissive attitudes towards the sensual worlds of infants and young children only changed significantly from the 1980s – not coincidentally in the contexts of contestations of the acute-pain model by women and ethnic minorities as well as debates within pro- and anti-abortion movements.

Finally, the social also adheres to the physiological body itself. This is the fourth advantage of conceiving of pain as a ‘way of naming an event’. The act of ‘naming’ influences bodily responses. Anthropologist William Reddy has called this ‘emotives’.^44^ Language does things to bodies. It acts upon them. This is another way of saying that the body-in-pain is not simply an entity awaiting social inscription (as in Wittgenstein's ‘the beetle in the box’ analogue or as implied in the ‘body as text’ metaphor) but is an active agent in both creating pain-events and, in turn, being created by them. The repeated recitation of particular way of naming a pain, for example, can affect the physiological body. Figurative languages can inform an individual's autonomic arousal, cardio-vascular responses and sensorimotor actions. Or, put in the language of the very different, humoural physiology, metaphors can affect whether blood freezes or gushes through the irritated, distended vessels of the body; they direct the ebb and flow of phlegm, black bile and yellow bile. Naming can instruct bodies how to respond. This concept of ‘retrojection’, or the means by which ways of naming pain are mapped back into the flesh, is important for any historian of the body. When a series of figurative languages or concepts for pain are repeated time and again from infancy, they become internalised and infused literally within the individual's body.^45^ Through retrojection, sufferers ‘infuse the imagery of cultural metaphors’ into their bodies, thus, feeling ‘the power of discourse within’.^46^

To conclude: sentience is socialised. It is a state of being, constituted within complex, temporal worlds. Pain is a type of event that involves sensation, cognition, affect and motivational aspects. Meanings, history, learning and expectations all influence ways of being-in-pain. As a type of event, pain is always meaningful to the person experiencing it. There is no pain-entity independent of the way it impinges on people's being-in-the-world. People often speak as though they ‘have’ a pain – and the contrasting ways they ‘have’ it is important in mapping changes over time – but the body-in-pain is a lived event. As a historically unstable practice, pain-events are constituted and reconstituted in relation to other practices, including language systems, social and environmental interactions and bodily comportment. Contestations to this ideological work is important. As Latham reminded his readers in the 1860s, the ‘things of life and feeling’ – that is, each person's *unique* encounter with suffering – are ‘different from all things in the world besides’.^47^

## Footnotes

1 Zafar H. Zaidi, ‘Hula-Hoop Syndrome’, Canadian Medical Association Journal, 80 (1959), 715–1613652012PMC1830882.

2 Friedrich Nietzsche, The Gay Science, trans. Walter Kaufmann (New York, 1974; 1st publ. 1882,), 249–50.

3 Silvia Camparesi, Barbara Bottalico and Giovanni Zamboni, ‘Can We Finally “See” Pain? Brain Imaging Techniques and Implications for the Law’, Journal of Consciousness Studies, 18, 9–10 (2011), 257–8.

4 René Descartes, ‘Meditations on First Philosophy’, trans. Elizabeth S. Haldane and G. R. T. Ross, ed. Enrique Chávez-Arvizo, Descartes: Key Philosophical Writings (Ware, 1997; 1st publ. 1641), 183, and René Descartes, Traité de l'homme (Paris, 1664), 27.

5 Christian Augustis Struve, *Asthenology: Or, the Art of Preserving Feeble Life; and of Supporting the Constitution under the Influence of Incurable Diseases*, trans. William Johnston (1801), 423.

6 E. Guttmann and W. Mayor-Gross, ‘The Psychology of Pain’, *The Lancet*, 20 Feb. 1943, 225.

7 For summaries, see Ronald Melzack and Patrick D. Wall, ‘Pain Mechanisms: A New Theory’, Science, 150, 3699 (1965), 971–9532081610.1126/science.150.3699.971, and Roselynne Rey, The History of Pain, trans. Louise Elliott Wallace (Cambridge, MA, 1995).

8 For the best discussion, see David B. Morris, The Culture of Pain (Berkeley, 1991), 9.

9 For a discussion, see Jan Frans Van Dijkhuizen, ‘Partakers of Pain: Religious Meanings of Pain in Early Modern England’, in The Sense of Suffering. Constructions of Physical Pain in Early Modern Culture, ed. Van Dijkhuizen and Karl A. E. Enenkel (Leiden, 2009), 189–219.

10 P. M. Latham, ‘General Remarks on the Practice of Medicine’, *British Medical Journal* (28 June 1862), 677.

11 *Ibid*.

12 *Ibid*.

13 Elaine Scarry, The Body in Pain. The Making and Unmaking of the World (New York, 1985), 4–5.

14 Geoffrey Galt Harpham, ‘Elaine Scarry and the Dream of Pain’, Salmagundi, 130/131 (2001), 208.

15 For an extensive discussion of metaphor and pain, see my article ‘Pain: Metaphor, Body, and Culture in Anglo-American Societies between the Eighteenth and Twentieth Centuries’, *Rethinking History* (forthcoming, 2014).

16 Paul Ricoeur, Oneself as Another, trans. Kathleen Blamey (Chicago, 1992), 132.

17 Ludwig Wittgenstein, Philosophical Investigations, trans. G. E. M. Anscombe (Oxford, 1953), 89.

18 *Ibid*., 188.

19 Guy Douglas, ‘Why Pains Are Not Mental Objects’, Philosophical Studies, 91, 2 (1998), 127–48.

20 *Ibid*., 127–48.

21 Latham, ‘General Remarks’, 677.

22 For a discussion, see my book *What It Means To Be Human: Reflections from 1791 to the Present* (2011).

23 Descartes, ‘Meditations on First Philosophy’, 183, and Descartes, *Traité de l'homme*, 27.

24 Edmund Burke, *The Works of the Right Hon. Edmund Burke, with a Biographical and Critical Introduction by Henry Rogers*, i (1837), 60.

25 Lt. Col. Henry K. Beecher, ‘Pain in Men Wounded in Battle’, Annals of Surgery, 123, 1 (1946), 96–10517858731PMC1803463.

26 Walter Freeman and James W. Watts, Psychosurgery. In the Treatment of Mental Disoerders and Intractable Pain, 2nd edn (Oxford, 1950), 550; J. W. Watts and Walter Freeman, ‘Psychosurgery for the Relief of Unbearable Pain’, Journal of the International College of Surgeons, 9 (1945), 67920279426; Everett G. Grantham and R. Glen Spurling, ‘Selective Lobotomy in the Treatment of Intractable Pain’, Annals of Surgery, 137, 5 (1953), 6021304111010.1097/00000658-195305000-00003PMC1802689; W. Tracey Haverfield and Christian Keedy, ‘Neurosurgical Procedures for the Relief of Intractable Pain’, Southern Medical Journal, 42, 12 (1949), 107710.1097/00007611-194912000-0001315407381; Mical Raz, ‘The Painless Brain Lobotomy, Psychiatry, and the Treatment of Chronic Pain and Terminal Illness’, Perspectives in Biology and Medicine, 52, 4 (2009), 56010.1353/pbm.0.012119855124.

27 Douglas, ‘Why Pains Are Not Mental Objects’, 127–48.

28 Rev. Joseph Townend, *Autobiography of the Rev. Joseph Townsend: With Reminiscences of his Missionary Labours in Australia*, 2nd edn (1869), 16–18.

29 ‘The Function of Physical Pain: Anæsthetics’, *Westminster Review*, 40, 1 (1871), 198–200 and 205.

30 For an extensive discussion, see my article ‘Pain: Metaphor, Body, and Culture in Anglo-American Societies between the Eighteenth and Twentieth Centuries’.

31 John Hervey, ‘An Account of my Own Constitution and Illness, with Some Rules for the Preservation of Health; for the Use of my Children’, in his *Some Materials Towards Memoirs of the Reign of King George II*, iii, ed. Romney Sedgwick (1931; 1st publ. 1731), 971.

32 *Ibid*.

33 For a discussion, see my *Stories of Pain: From Prayer to Painkillers* (Oxford, forthcoming, 2014).

34 Rachel Betts, *Memoir of the Last Illness and Death of Rachel Betts* (1834), 22.

35 Ronald Melzack, ‘The Perception of Pain’, in Physiological Psychology, ed. Richard F. Thompson (San Francisco, 1971), 223.

36 Martin S. Pernick, A Calculus of Suffering. Pain, Professionalism, and Anesthesia in Nineteenth Century America (New York, 1985).

37 For an extended discussion, see my ‘Pain Sensitivity: An Unnatural History from 1800 to 1965’, *Journal of Medical Humanities* (forthcoming, 2013).

38 This is addressed in great detail in my *Stories of Pain*.

39 Michael Underwood, *A Treatise on the Diseases of Children, with Directions for the Management of Infants from the Birth; especially Such as Are Brought up by Hand* (1784), 4.

40 Pye Henry Chavasse, Advice to a Mother on the Management of her Children, and on the Treatment on [sic] the Moment of Some of their More Pressing Illnesses and Accidents, 9th edn (Philadelphia, 1868), 70.

41 Paul Flechsig's lecture, summarized by Frederick W. Mott, ‘Cerebral Development and Function’, British Medical Journal, 1, 3145 (1921), 529.

42 Max Thorek, Modern Surgical Technique, iii (Philadelphia, 1938), 2012.

43 Joann [*sic*] M. Eland and Jane E. Anderson's chapter in *Pain. A Sourcebook for Nurses and Other Health Professionals*, ed. Ada Jacox (Boston, MA, 1977), 453–76.

44 William M. Reddy, The Navigation of Feeling: A Framework for the History of Emotions (New York, 2001).

45 I develop this argument in ‘Pain: Metaphor, Body, and Culture in Anglo-American Society between the Eighteenth and Twentieth Centuries’.

46 Michael Kimmel, ‘Properties of Cultural Embodiment: Lessons from the Anthropology of the Body’, in Body, Language, and Mind, ii: *Sociocultural Stuatedness*, ed. Rosleyn M. Frank, René Dirven, Tom Ziemke, and Enriquè Bernárdez (New York, 2008), 99 and 101.

47 Latham, ‘General Remarks’, 677.

